# Nitrogen availability prevents oxidative effects of salinity on wheat growth and photosynthesis by up-regulating the antioxidants and osmolytes metabolism, and secondary metabolite accumulation

**DOI:** 10.1186/s12870-019-2085-3

**Published:** 2019-11-08

**Authors:** Mohammad Abass Ahanger, Cheng Qin, Naheeda Begum, Qi Maodong, Xu Xue Dong, Mohamed El-Esawi, Mohamed A. El-Sheikh, Abdulrahman A. Alatar, Lixin Zhang

**Affiliations:** 1 College of Life Sciences, Northwest A&F University, Yangling, Shaanxi, China; 2 Botany Department, Faculty of Science, Tanta University, Tanta, Egypt; 3 Sainsbury Laboratory, University of Cambridge, Cambridge, UK; 4 Botany and Microbiology Department, College of Science, King Saud University, Riyadh, 11451 Saudi Arabia; 5 Botany Department, Faculty of Science, Damanhour University, Damanhour, Egypt

**Keywords:** Nitrogen, Antioxidants, AsA-GSH cycle, Oxidative damage, Salinity, *Triticum aestivum*

## Abstract

**Background:**

Salinity is one of the damaging abiotic stress factor. Proper management techniques have been proposed to considerably lower the intensity of salinity on crop growth and productivity. Therefore experiments were conducted to assess the role of improved nitrogen (N) supplementation on the growth and salinity stress tolerance in wheat by analyzing the antioxidants, osmolytes and secondary metabolites.

**Results:**

Salinity (100 mM NaCl) stress imparted deleterious effects on the chlorophyll and carotenoid synthesis as well as the photosynthetic efficiency. N supplementation resulted in increased photosynthetic rate, stomatal conductance and internal CO_2_ concentration with effects being much obvious in seedlings treated with higher N dose. Under non-saline conditions at both N levels, protease and lipoxygenase activity reduced significantly reflecting in reduced oxidative damage. Such effects were accompanied by reduced generation of toxic radicals like hydrogen peroxide and superoxide, and lipid peroxidation in N supplemented seedlings. Antioxidant defence system was up-regulated under saline and non-saline growth conditions due to N supplementation leading to protection of major cellular processes like photosynthesis, membrane structure and function, and mineral assimilation. Increased osmolyte and secondary metabolite accumulation, and redox components in N supplemented plants regulated the ROS metabolism and NaCl tolerance by further strengthening the antioxidant mechanisms.

**Conclusions:**

Findings of present study suggest that N availability regulated the salinity tolerance by reducing Na uptake and strengthening the key tolerance mechanisms.

## Background

Plants are continuously confronted by variety of environmental stresses resulting in significant growth retardation and yield reduction. Salinity stress is considered as one of the damaging abiotic stress factor affecting metabolism and productivity of crop plants allover the globe [1, 2]. Increasing salinity has been considered as global threat to food security causing significant conversion of agricultural arable land into unproductive waste land. Salinity affects mineral uptake and assimilation, enzyme activity, photosynthesis, protein expression and hormone metabolism [2, 3]. Excess salt concentrations in growth medium induces osmotic and ionic stress resulting in occurrence of obvious growth changes including reduced leaf area, necrosis and abscission [4, 5]. Tolerance to salinity is a complex trait involving several physiological, biochemical, molecular and gene networks [6]. Salinity disturbs the ionic balance resulting in reduction of water content, and oxidative damage due to accumulation of excess reactive oxygen species (ROS) leading to peroxidation of lipids [3, 7]. It is essential to identify the physio-biochemical and molecular attributes for enhancing the salinity tolerance [6]. Salinity stress has been reported to affect the uptake and metabolism of essential elements like N, P, K, S and Ca leading to significant alterations in the photosynthetic efficiency and the tolerance mechanisms involved [1, 7]. Among the most deleterious products of saline environment are included the ROS like superoxide, hydrogen peroxide, peroxide and hydroxyl radical [1, 8]. To avoid the salinity mediated growth restrictions plants up-regulate certain indigenously occurring tolerance mechanisms like antioxidant system, osmolyte and secondary metabolite accumulation [6, 9]. Salinity induced increase in ROS results in oxidative damage to important molecules including proteins, lipids, nucleic acids etc. Increased ROS accumulation hampers redox homeostasis declining the photosynthetic efficiency [7], and nutrient and osmolyte metabolism [1]. For assuaging the salinity mediated ROS-induced deleterious effects on growth the antioxidant system and osmoregulatory components are up-regulated [2]. In addition to this salt exclusion at root and vacuole level is considered as an important key mechanism regulating tolerance in plants [10, 11]. It has been reported that plants displaying greater antioxidant and osmolyte metabolism in addition of the selective accumulation of mineral ions exhibit increased tolerance to salinity [2, 7]. Every cellular compartment has its own set of ROS-producing and -neutralising pathways for maintaining the steady-state levels of ROS and the redox state, thereby giving rise to distinct ROS signatures in different cellular compartments [1, 8, 12]. It is believed that different ROS signatures determine the stress acclimation specificity by mediating systemic signalling. Antioxidant system is constituted of both enzymatic and non-enzymatic components [12, 13]. Compatible osmolytes like proline, glycine betaine and sugars assist the antioxidant system in neutralising the excess ROS. Osmolytes act as nutrient and metabolite signalling molecules activating specific or hormone crosstalk transduction pathways and modify gene expression and proteomic patterns [13]. Nitrogen (N), a macroelement which is actively involved in regulation of enzyme activity, photosynthesis, protein synthesis, antioxidant and osmolyte metabolism [14–16]. N forms the component of major molecules including nucleic acids, proteins, chlorophylls etc. and its deficient availability results in oxidative damage to membranes, photosynthetic inhibition and impeded nutrient uptake [17, 18]. N availability regulates the synthesis of hormones, osmolytes [16, 19], secondary metabolites [20] and the activity of antioxidant system [15]. Earlier Ahanger and Agarwal [1, 21] have reported that up-regulated antioxidant and osmolyte metabolism prevents salinity and water stress mediated growth inhibition by protecting nitrogen and secondary metabolite metabolism. However, the role of N availability in regulation of antioxidant, osmolyte and secondary metabolism under salinity stress remains to be least researched area. It is with this backdrop we hypothesized that (a) whether N supplementation modulates antioxidant, osmolyte and secondary metabolite metabolism for enhancing the salinity tolerance, and (b) the effectivity of improved N supplementation in the alleviation of salinity mediated changes in growth and physio-biochemical attributes.

## Results

### N supplementation reduced Na accumulation and improved K uptake

Results regarding the effect of N availability on the uptake of Na and K are shown in Table 1. Relative to control, accumulation of Na increased by 56.94% due to NaCl treatment which was declined by 40.36 and 56.15% due to supplementation of N at 50 and 100 mg kg^− 1^ soil (N1 and N2) respectively over the NaCl stressed counterparts. Under normal conditions, supplementation of N significantly reduced Na accumulation with maximal decline of 65.05% observed with 100 mg kg^− 1^ (N2). K uptake was significantly improved by increasing N supplementation attaining maximal increase of 32.74% with N2. Nitrogen supplementation proved beneficial in reducing the NaCl mediated decline in K with percent amelioration of 18.50 and 34.63% at N1 and N2 respectively over the NaCl stressed plants (Table 1).

**Table 1:** Effect of nitrogen supplementation on the uptake of sodium, potassium, nitrogen and nitrate reductase activity in *Triticum aestivum* L subjected to salinity stress. Data is mean (±SE) of three replicates. Values followed by different letters are significantly different at *P* < 0.05

	Control	NaCl	N1	N2	NaCl + N1	NaCl + N2
Na (mg g^− 1^ DW)	5.98 ± 0.54c	13.89 ± 0.92a	3.40 ± 0.17d	2.09 ± 0.15de	9.90 ± 0.81b	6.09 ± 0.59c
K (mg g^− 1^ DW)	22.78 ± 1.8c	13.87 ± 1.01e	26.09 ± 2.1b	33.87 ± 2.4a	17.02 ± 1.12d	21.22 ± 1.9c

### Pigment synthesis and photosynthesis improved due to N supplementation under NaCl stress

Salinity stress reduced the synthesis of chlorophyll and carotenoid pigments resulting in declined photosynthetic rate. Total chlorophyll, carotenoids, photosynthetic rate, stomatal conductance, intercellular CO_2_ concentration and transpiration rate was observed to increase with N supplementation reaching to maximal increase of 38.71, 32.08, 51.47, 33.71, 29.37 and 33.47% over the control plants with N2. Maximal amelioration of salinity mediated decline was observed with N2 with 47.36% for total chlorophyll, 56.39% for carotenoids, 54.31% for photosynthetic rate, 30.91% for stomatal conductance, 33.64% for intercellular CO_2_ concentration and 42.81% for transpiration rate over the NaCl stressed plants (Table 2).

**Table 2:** Effect of nitrogen supplementation on the total chlorophyll, carotenoids and gas exchange parameters in *Triticum aestivum* L subjected to salinity stress. Data is mean (±SE) of three replicates. Values followed by different letters are significantly different at *P* < 0.05

	Control	NaCl	N1	N2	NaCl + N1	NaCl + N2
Total Chlorophyll (mg g^− 1^ FW)	1.621 ± 0.045c	0.9105 ± 0.016e	2.134 ± 0.131b	2.645 ± 0.142a	1.326 ± 0.0532d	1.730 ± 0.050c
Carotenoids (mg g^− 1^ FW)	0.4620 ± 0.011c	0.3011 ± 0.010e	0.5123 ± 0.013b	0.6803 ± 0.012a	0.3982 ± 0.011d	0.4709 ± 0.014c
Photosynthetic rate (μmol CO_2_ m^−2^ s^− 1^)	11.81 ± 0.54d	7.73 ± 0.51e	17.21 ± 1.01b	24.34 ± 1.21a	13.33 ± 0.45c	16.92 ± 1.001b
Net intercellular CO_2_ (μmol m^− 2^ s^− 1^)	226.1 ± 7.12d	176.1 ± 6.15e	298.3 ± 9.15b	341.1 ± 9.98a	222.9 ± 6.99d	254.9 ± 8.012c
Stomatal conductance (mmol m^− 2^ s^− 1^)	312.1 ± 8.52d	246.3 ± 7.01e	398.1 ± 10.21b	441.9 ± 15.01a	314.6 ± 8.11d	371.2 ± 9.77bc
Transpiration rate (mmol H_2_O m^− 2^ s^− 1^)	3.14 ± 0.10c	1.72 ± 0.011e	4.14 ± 0.14b	4.72 ± 0.13a	2.33 ± 0.031d	3.008 ± 0.21c

### N availability induces synthesis of osmolytes under salinity stress

**Fig. 1:**
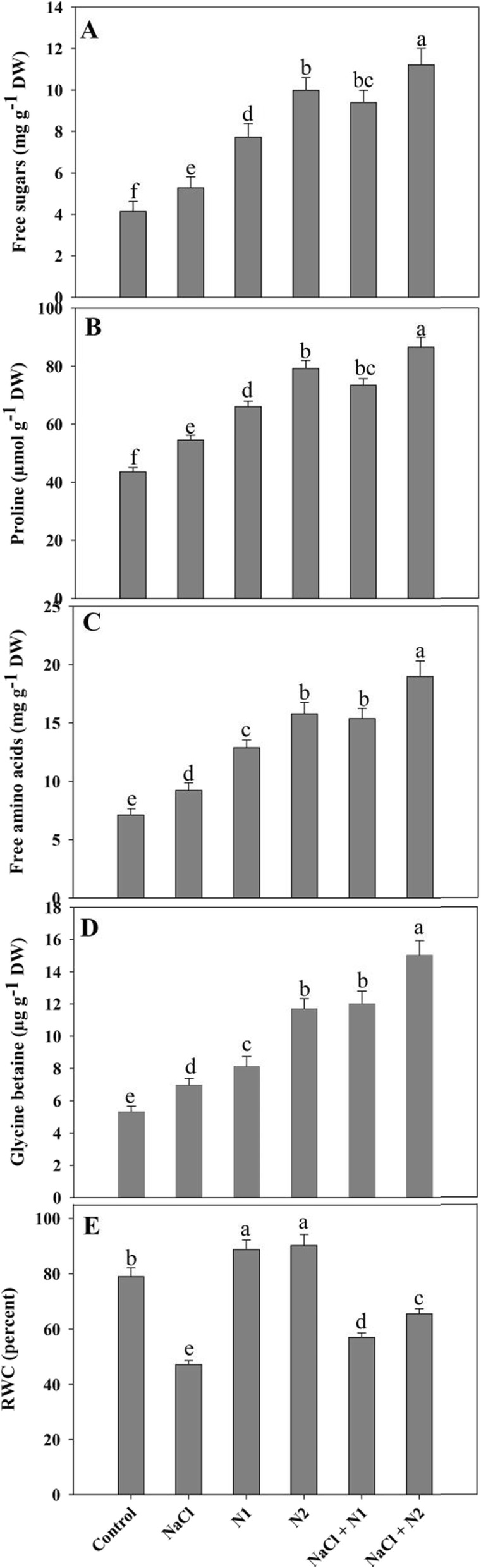
Effect of nitrogen (50 and 100 mg kg^− 1^ soil) supplementation on the content of (**a**) free sugars, (**b**) free proline, (**c**) free amino acids, (**d**) glycine betaine and (**e**) relative water content in *Triticum aestivum* L subjected to salinity stress. Data is mean (±SE) of three replicates, bars denoted by different letters are significantly different at *P* < 0.05

Proline, free amino acids, glycine betaine and sugars increased due to supplementation of N and maximal accumulation was observed with higher N dose. Relative to control, proline, free amino acids, glycine betaine and sugars increased by 34.07, 44.72, 46.57 and 34.52% due to supplementation of 50 N and by 44.95, 54.87, 54.52 and 58.61% due to 100 N. Maximal percent increase of 49.61% for proline, 62.50% for free amino acids, 64.03% for glycine betaine and 63.19% for sugars was observed in NaCl + 100 N over the control (Fig. 1) and these values were much higher than the NaCl stressed ones.

### Increased N application reduced oxidative stress

**Fig. 2:**
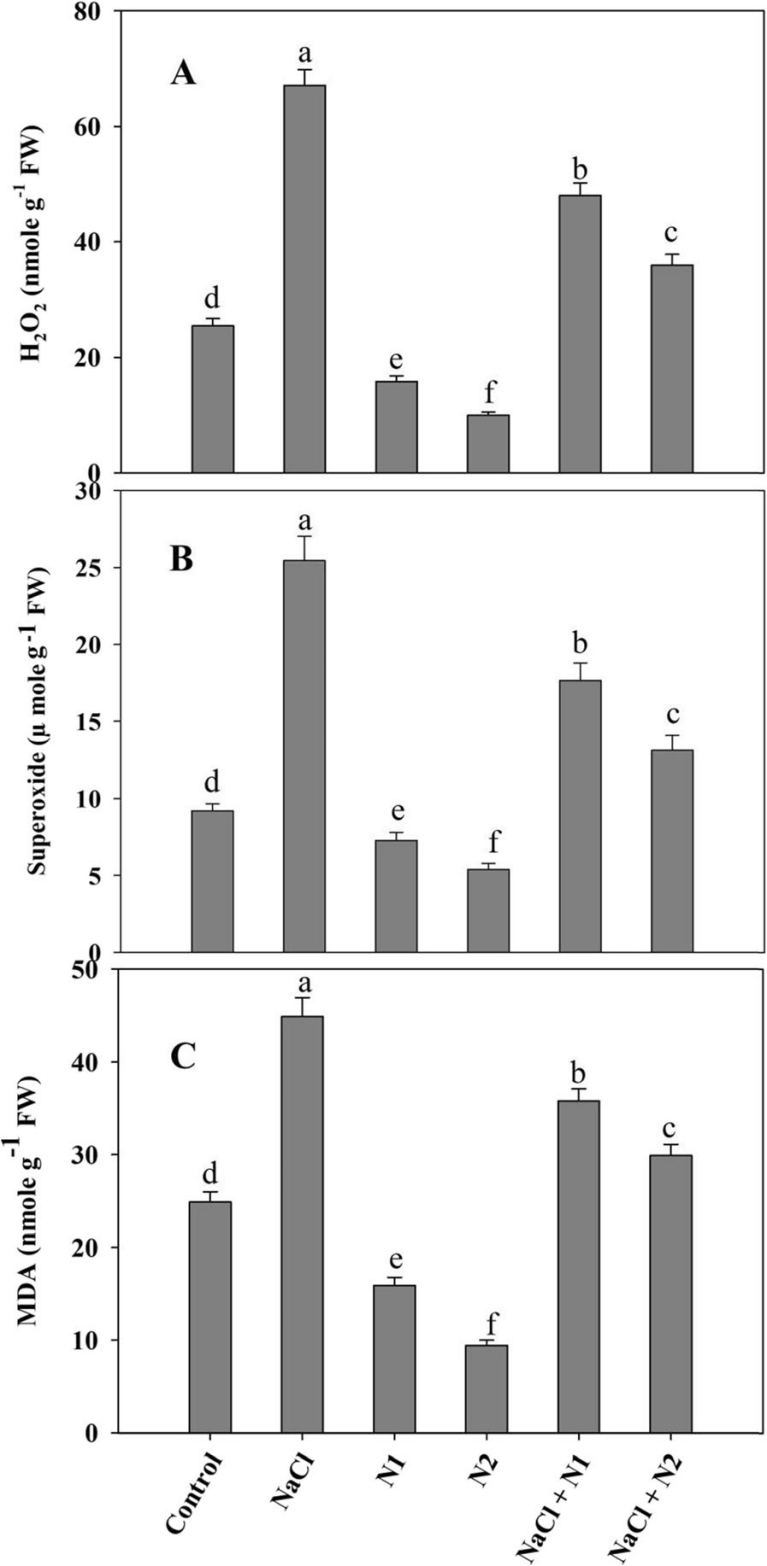
Effect of nitrogen (50 and 100 mg kg^− 1^ soil) supplementation on the (**a**) hydrogen peroxide, (**b**) superoxide and (**c**) lipid peroxidation in *Triticum aestivum* L subjected to salinity stress. Data is mean (±SE) of three replicates, bars denoted by different letters are significantly different at *P* < 0.05

**Fig. 3:**
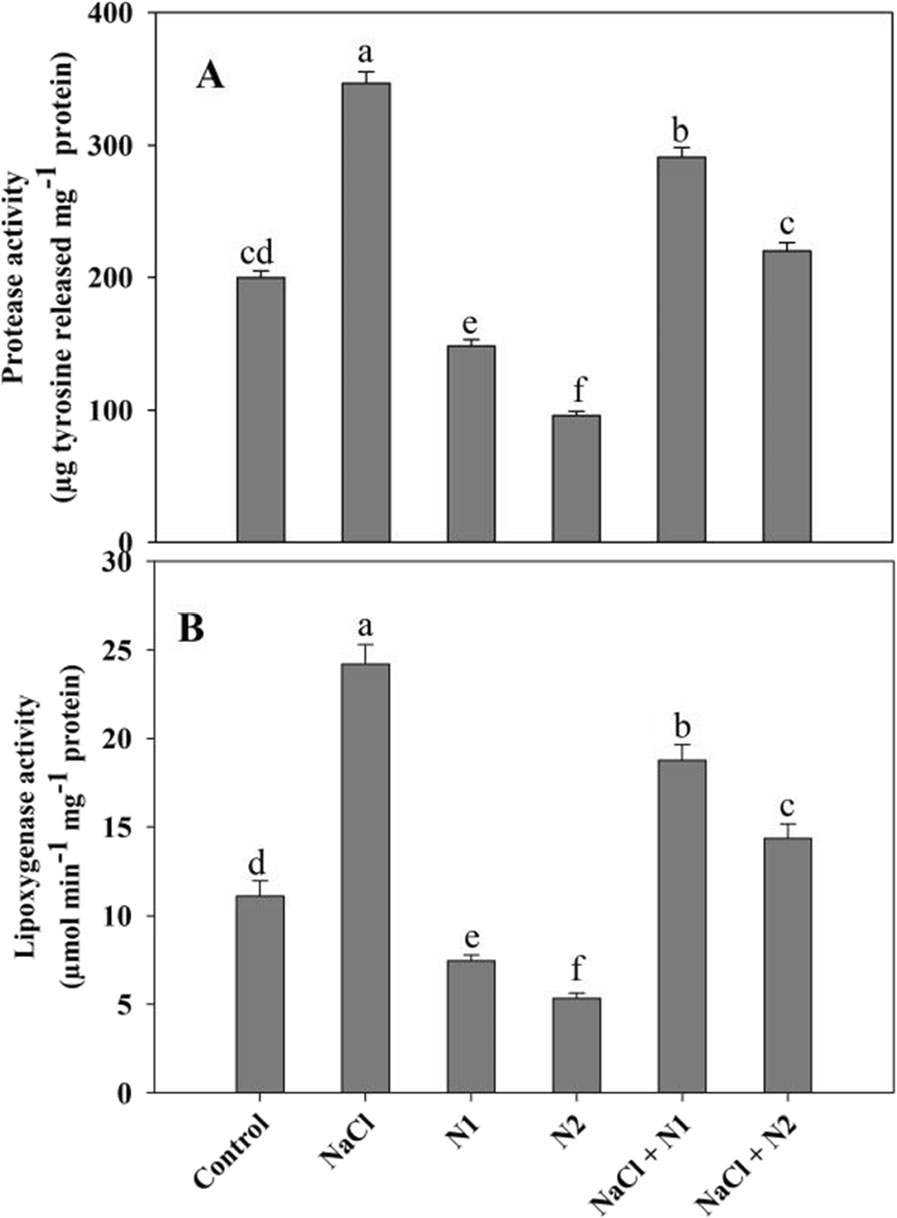
Effect of nitrogen (50 and 100 mg kg^− 1^ soil) supplementation on the (**a**) protease and (**b**) lipoxygenase activity in *Triticum aestivum* L subjected to salinity stress. Data is mean (±SE) of three replicates, bars denoted by different letters are significantly different at *P* < 0.05

Seedlings exposed to salinity exhibited increased generation of free radicals like H_2_O_2_ and O_2_^−^ over the control and N supplemented ones. Percent increase in H_2_O_2_ and O_2_^−^ due to NaCl was 62.11 and 63.78% respectively causing 44.60% increase in lipid peroxidation over the control. Relative to control, N supplementation significantly declined the generation of H_2_O_2_ (2.5 fold) and O_2_^−^ (1.7 fold) causing 62.01% decline in lipid peroxidation. Supplementation of N2 to NaCl stressed plants maximally ameliorated the generation of H_2_O_2_ and O_2_^−^ resulting in 33.42% decline in lipid peroxidation as compared to NaCl stressed ones (Fig. 2a-c). N fed seedlings showed apparent decline in the activities of protease and lipoxygenase over the control as well as NaCl stressed ones with maximal decline observed with higher N. Relative to control, protease and lypoxygenase increased by 1.73 and 2.17 folds in NaCl stressed plants, however supplementation of N maintained the effect even under NaCl conditions. At 100 N (N2), protease and lypoxygenase decreased by 52.00 and 51.97% respectively, and NaCl + N2 treated plants exhibited a decline of 36.54 and 40.72% over the NaCl stressed counterparts (Fig. 3a-b).

### N supplementation up-regulates the antioxidant system

**Fig. 4:**
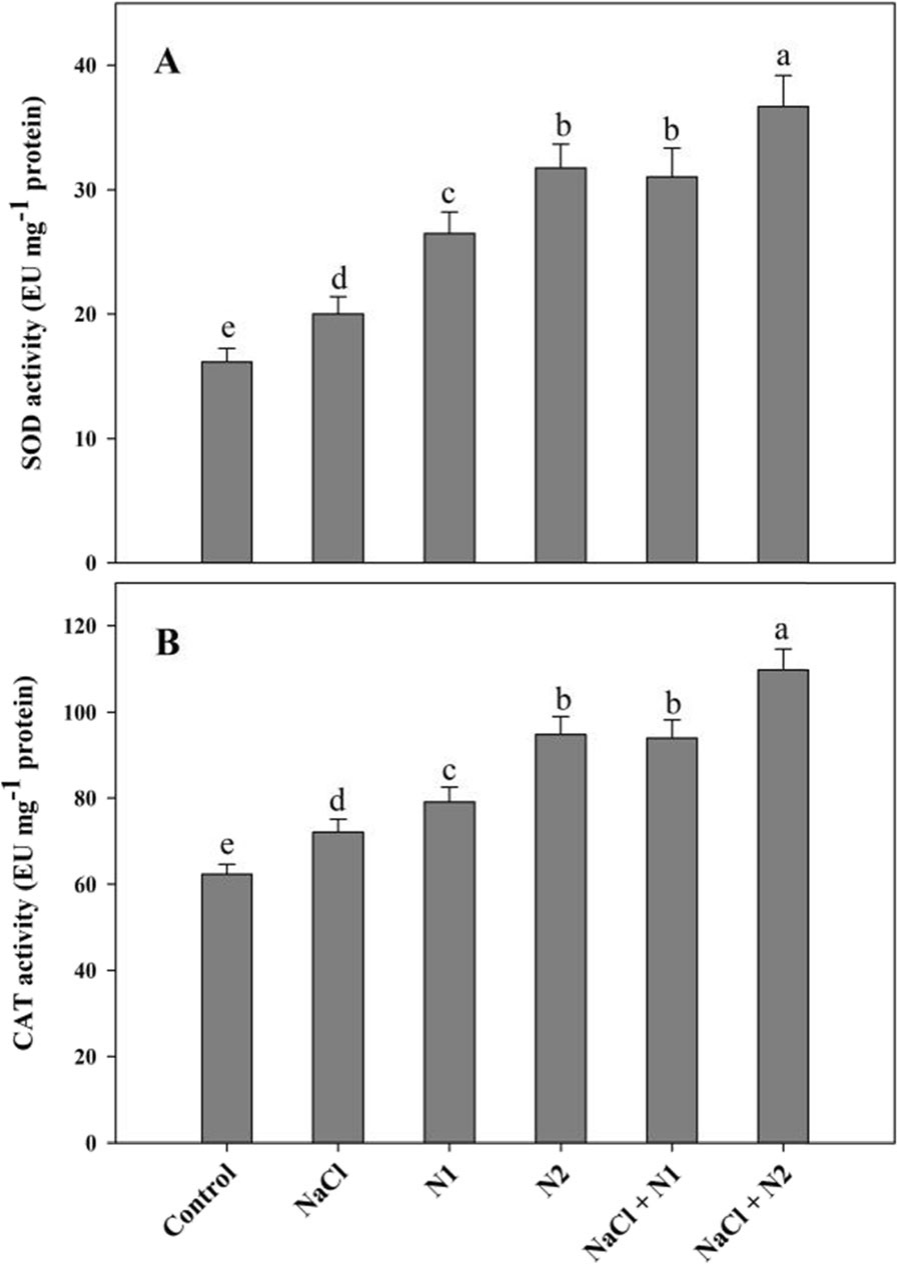
Effect of nitrogen (50 and 100 mg kg^− 1^ soil) supplementation on the (**a**) superoxide dismutase and (**b**) catalase activity in *Triticum aestivum* L subjected to salinity stress. Data is mean (±SE) of three replicates, bars denoted by different letters are significantly different at *P* < 0.05

**Fig. 5:**
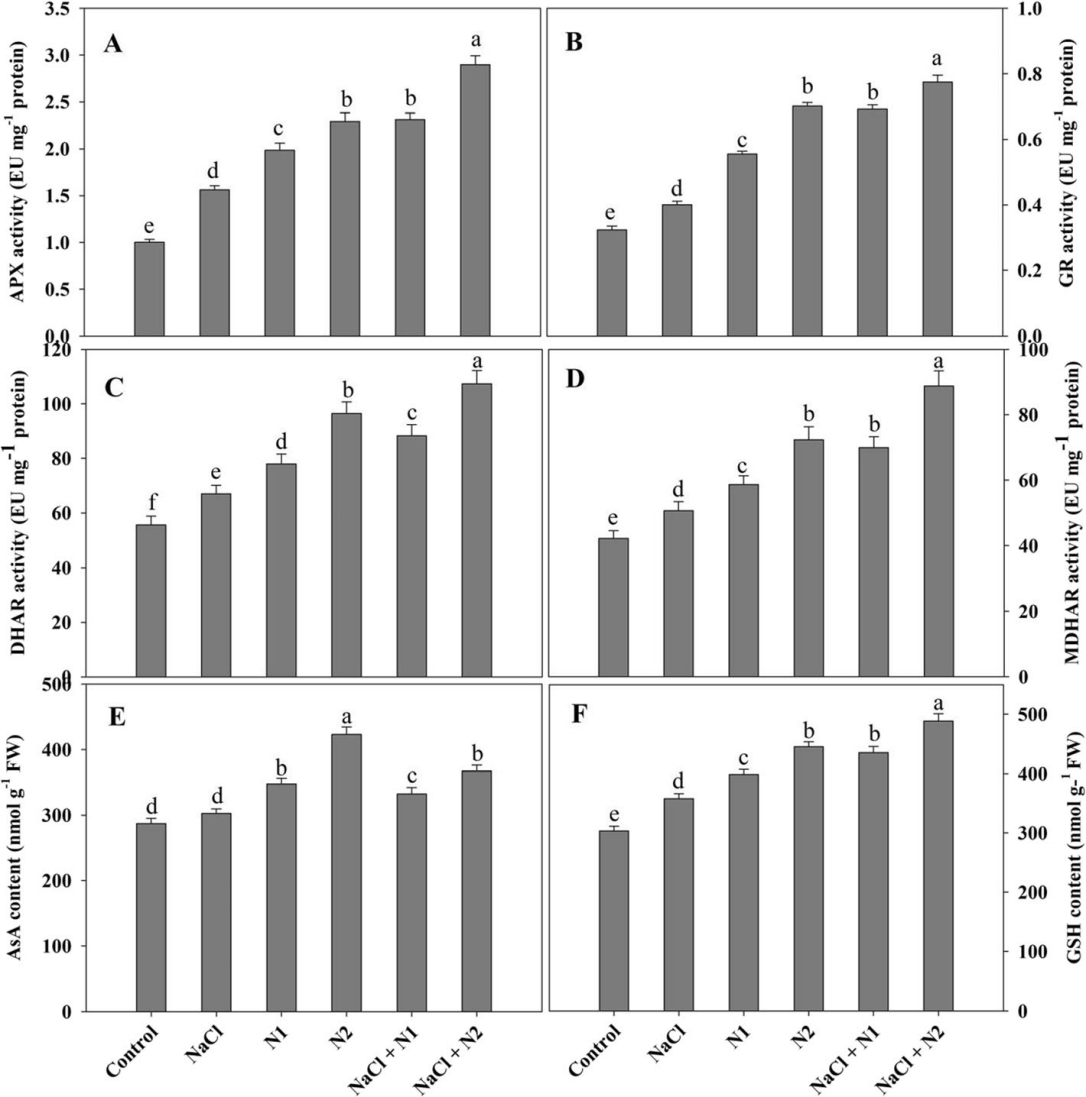
Effect of nitrogen (50 and 100 mg kg^− 1^ soil) supplementation on the (**a**) ascorbate peroxidase, (**b**) glutathione reductase, (**c**) dehydroascorbate reductase, (**d**) monodehydroascorbate reductase activity and content of (**e**) ascorbate and (**f**) reduced glutathione in *Triticum aestivum* L subjected to salinity stress. Data is mean (±SE) of three replicates, bars denoted by different letters are significantly different at *P* < 0.05

**Fig. 6:**
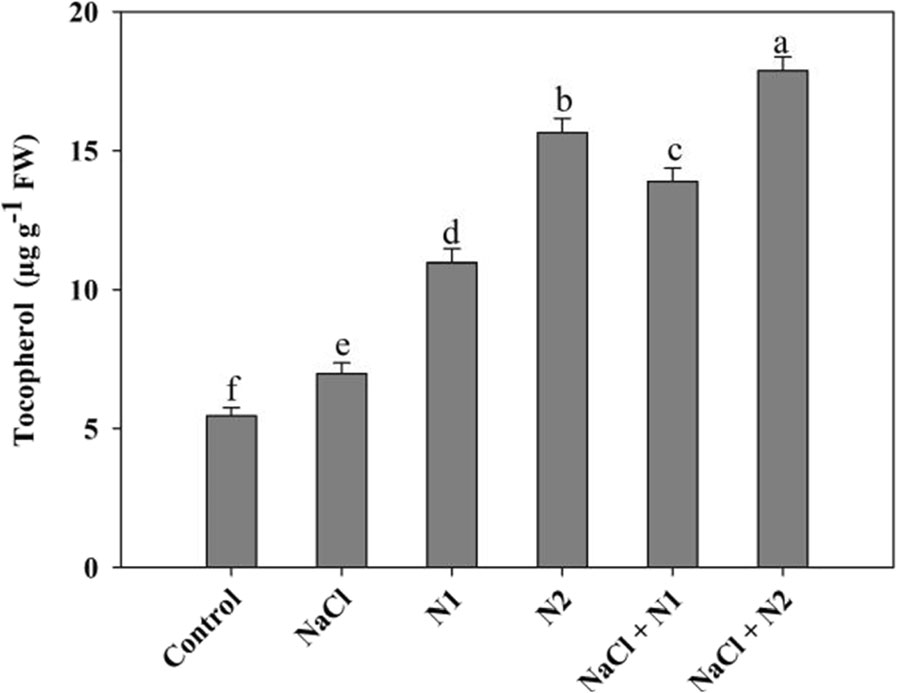
Effect of nitrogen (50 and 100 mg kg^− 1^ soil) supplementation on the tocopherol content in *Triticum aestivum* L subjected to salinity stress. Data is mean (±SE) of three replicates, bars denoted by different letters are significantly different at *P* < 0.05

Results revealed that availability of N significantly affected the antioxidant system by up-regulating the activity of SOD, CAT, APX, GR, MDHAR, DHAR and the synthesis of AsA and GSH. Relative to control, under normal conditions maximal increase in SOD (1.96 fold), CAT (1.52 fold), APX (2.28 fold), GR (2.16 fold), MDHAR (1.71 fold), DHAR (1.73 fold), AsA (1.47 fold), and GSH (1.46 fold) was observed with 100 N (N2). Though NaCl stress triggered the activity of antioxidant enzymes however, N supplemented seedlings exhibited maximal activity with increase of 2.27 fold for SOD, 1.76 fold for CAT, 2.89 fold for APX, 2.39 fold for GR, 2.10 fold for MDHAR, 1.93 fold for DHAR and 1.61 fold for GSH in NaCl + N2 treated seedlings (Figs. 4 and 5). Tocopherol content also exhibited apparent increase with N supplementation. Relative to control, tocopherol increased by 50.27 and 65.17% due to N1 and N2 supplementation respectively. Applied N (N100) maintained its effect on the tocopherol under NaCl conditions leading to an enhancement of 60.99% over the NaCl stressed pants (Fig. 6).

### Phenols and flavonoids increased in N supplemented seedlings

**Fig. 7:**
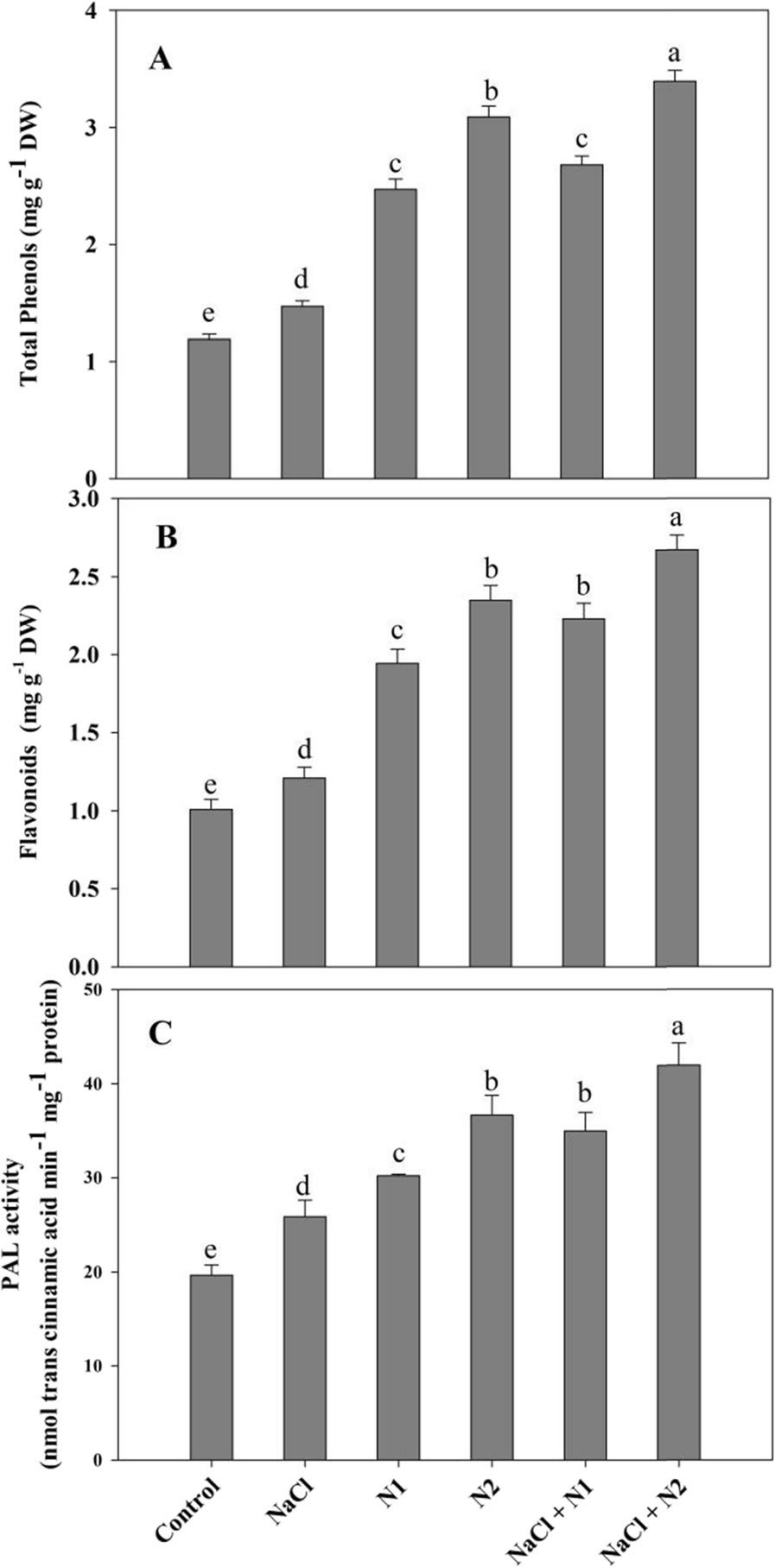
Effect of nitrogen (50 and 100 mg kg^− 1^ soil) supplementation on the content of (**a**) total phenol and (**b**) flavonoids, and the activity of (**c**) phenylalanine ammonia lyase in *Triticum aestivum* L subjected to salinity stress. Data is mean (±SE) of three replicates, bars denoted by different letters are significantly different at *P* < 0.05

The content of phenols and flavonoids was maximum in N supplemented seedlings under normal as well as NaCl stressed conditions. Percent increase in phenol and flavonoids was 19.04 and 16.54% due to NaCl stress and was further increased due to application of N attaining maximal values with NaCl + 100 N over control plants. Moreover N availability significantly affected activity of PAL imparting 1.53 and 1.86 fold increase with 50 and 100 N respectively, and reaching to maximum of 53.14% with NaCl + 100 N (Fig. 7).

### NR activity and N content increased with supplementation of N

**Fig. 8:**
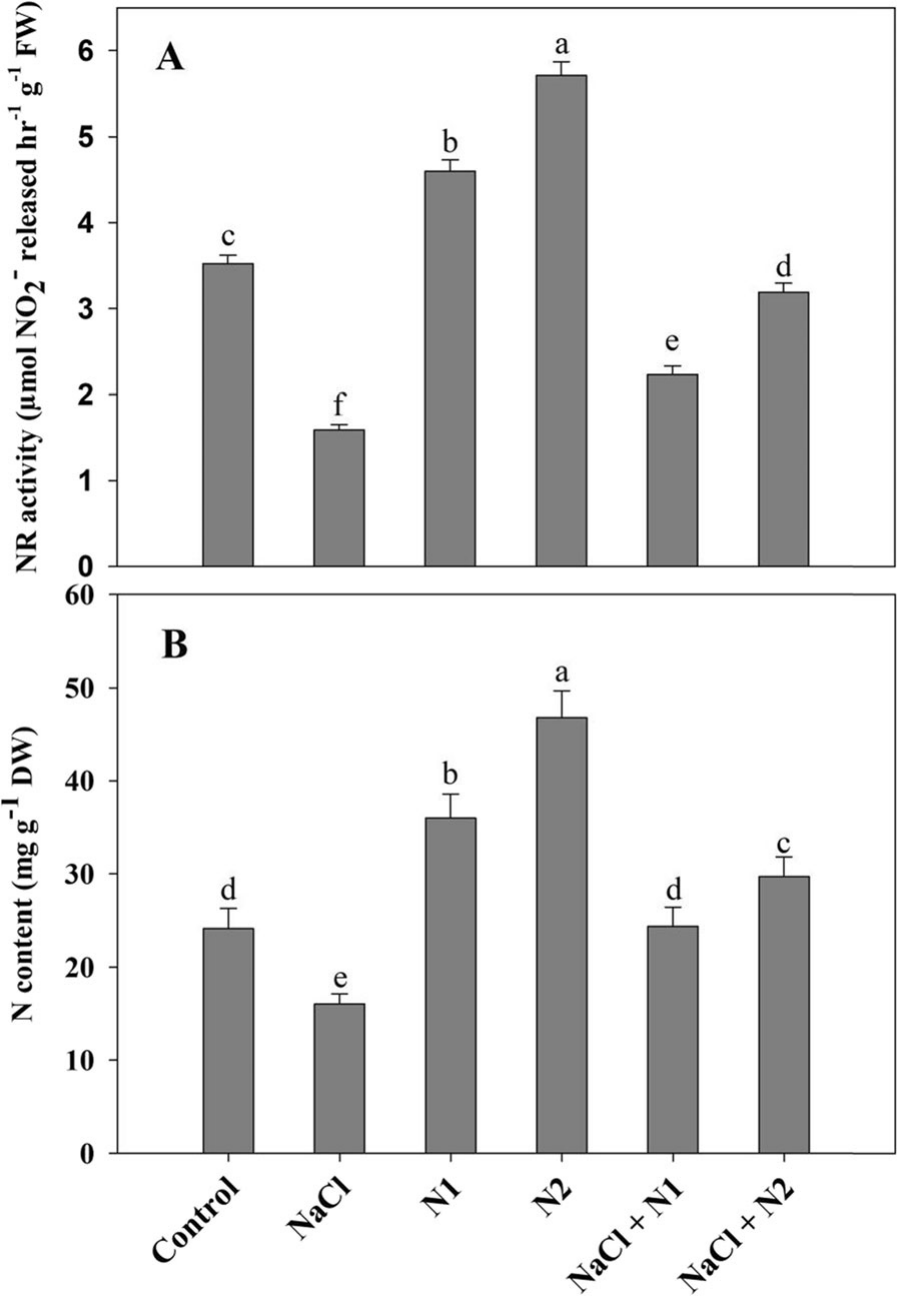
Effect of nitrogen (50 and 100 mg kg^− 1^ soil) supplementation on the activity of (**a**) nitrate reductase and (**b**) nitrogen content in *Triticum aestivum* L subjected to salinity stress. Data is mean (±SE) of three replicates, bars denoted by different letters are significantly different at *P* < 0.05

N supplementation significantly increased the activity of NR over the control plants and also ameliorated the decline caused by NaCl. Relative to control, NaCl caused a decline of 54.82% for NR and 33.62% for N content. Activity of NR was enhanced by 1.30 and 1.62 fold with 50 and 100 N resulting in 1.49 and 1.93 fold increase leaf N content. Maximal amelioration of 50.15 and 46.02% in NR activity and N content was observed in NaCl + 100 N treated seedlings over the NaCl stressed counterparts (Fig. 8).

## Discussion

Salinity has been one of the major problems of the sustainable agricultural productivity due to its toxic effects on the metabolism of the crop plants [22]. The accumulation of Na and other toxic ions alter the physiological stability of plant cells leading to considerable damage to their structural and functional stability [23]. Therefore to mitigate or lessen the deleterious effects of excess Na plants tend to reduce the salt-induced ionic toxicity by improving the strategies aimed at improving the salt tolerance via minimizing Na^+^ buildup in photosynthetic organs [24], improving K^+^ levels resulting in enhanced K^+^/Na^+^ ratio [1, 25]. This selective ion transport leading to Na^+^ homeostasis has been reported to be driven by several metabolic constituents like ROS [26], phytohormones [3], compatible osmolyte accumulation [1], and mineral uptake and assimilation [27]. In present study we studied the role of one of the key mineral (N) availability on the regulation of salt tolerance through the modifications at physiological and biochemical levels. It was observed that N availability significantly affected the growth of wheat seedlings by restricting the uptake of Na with concomitant improvement in K. N deficiency declines the cellular division hence reducing the cell number and tissue proliferation [28]. Juppner et al. [29] have demonstrated that accumulation of N containing compounds regulates the cell cycle progression, growth and biomass accumulation under the control of kinase activity. Excess Na is effluxed via the Na/H exchangers (NHX) into vacuole or by SOS1 proteins at the root levels [30]. Improved activities of transport proteins involved in active compartmentation and sequestration of Na ions significantly contributes to salinity stress tolerance in plants [31] and in present study N availability mediated decline in Na accumulation may have resulted due to increased transport protein expression preventing Na uptake at root level. Increased N supplementation resulted in significant increase in the uptake of N and K accompanied by reduced Na accumulation. Earlier the ameliorative role of increased N [19] and K [1] supplementation under salinity stress have been ascribed to their potentiality to prevent Na accumulation. K is ubiquitous for plant growth and development, stress mitigation, enzyme activity and osmolyte production [1, 12, 21] and additionally K itself is an important inorganic osmolyte [1, 32]. N supplementation improved the activity of NR facilitating its quick conversion into N precursors for synthesis of amino acids and hence proteins. Greater N availability directly affects the photosynthetic process by influencing the Rubisco synthesis [33]. Recently Iqbal et al. [19] have also demonstrated increased Rubisco activity in N supplemented seedlings causing significant amelioration of salinity mediated decline in photosynthesis. N supplementation improves the expression of small as well as large sub unit of Rubisco [34]. In present study increasing N supplementation imparted apparent enhancement in pigment synthesis and the photosynthetic efficiency and it was evident that N supplemented seedlings exhibited less decline due to NaCl treatment. Salinity stress reduces photosynthetic efficiency through its deleterious effects on the synthesis of chlorophylls and Rubisco protein [19]. Photosynthetic efficiency is corelated with N and Rubisco concentration and internal CO_2_ concentrations [35], and in present study higher N proved much affective in photosynthetic regulation. Further studies are required to unravel the exact mechanisms. N supplementation significantly increased the content of compatible solutes including proline, glycine betaine, sugars and free amino acids than the control as well as salt stressed counterparts. Increased N supplementation proved much affective in improving the osmolyte accumulation reflecting in maintenance of growth and hence the amelioration of salinity stress mediated growth retardation. In addition of their involvement in the maintenance of tissue water content osmolytes protect major cellular functioning by mediating stress signalling leading to activation of downstream tolerance mechanisms [36–38]. Osmolytes including sugars and amino acids like proline serve as ROS scavengers [39, 40]. N mediated increased accumulation of compatible solutes prevent oxidative damage induced growth restrictions by protecting the photosynthetic electron transport through ROS elimination and redox homeostasis. Increased accumulation of proline and glycine betaine significantly prevent the salinity induced photosynthetic inhibition [7]. Tissue concentration of compatible solutes is maintained either by irreversible synthesis of the compounds or by a combination of synthesis and degradation, and accumulation of osmolytes is proportional to the external osmolarity [6]. Osmolytes protect the structure and the osmotic balance of cells by maintaining the water influx [37]. Proline, sugars and glycine betaine protect the carboxylase activity of Rubisco [41, 42] and hence N supplementation induced increase in their accumulation may have contributed to better photosynthetic efficiency. Martino et al. [43] has demonstrated that accumulated glycine betaine and free amino acids constitute the maximal N containing osmolytes. Therefore increased N supplementation prevented the salinity mediated decline in growth of wheat seedlings by improving water content as well as the photosynthetic efficiency. Accumulated osmolytes assist in quick growth recovery after stress release [1, 21, 44]. N availability significantly improved the antioxidant metabolism by up-regulating the activity of key antioxidant enzymes and the contents of non-enzymatic components. N mediated improved antioxidant potential resulted in alleviation of salinity induced oxidative damage to a significant level. SOD is indespensible for the dismutation of superoxide radicals for preventing the damage to photosynthetic machinery. Increased antioxidant functioning reduces the stress mediated damage to membranes, proteins, nucleic acids and hence maintaining the functional stability [2, 8]. Earlier it has been reported that increased SOD, CAT and the AsA-GSH functioning prevent the stress triggered oxidative damage [2, 45, 46]. However the reports describing the N availability induced regulation of antioxidant system are scanty and further studies can be handy in understanding the actual underlying mechanisms. Salinity stress reduces the redox homeostasis thereby hampering the redox dependent cellular functions like electron transport and energy generation [7, 47]. Up-regulated AsA-GSH cycle functioning prevents the excess generation of toxic radicals by maintaining the NADP levels in chloroplast. Ascorbic acid, glutathione and tocopherol are the low molecular weight redox buffers and can also interact with numerous other cellular components. In addition of their obvious role in defense, as enzyme cofactors, these redox components regulate growth and development of plants by regulating key processes like mitosis, cell elongation, senescence etc. [47, 48]. Tocopherol preferably neutralizes singlet oxygen in addition of other ROS [48, 49]. Enzymes of AsA-GSH pathway contribute to efficient H_2_O_2_ removal thereby reducing the chances of radical formation through maintainence of redox homeostasis and hence preventing the oxidative damage to key cellular processes like electron transport [50]. N mediated up-regulation of antioxidant system was accompanied by reduced lipoxygenase and protease activity reflecting in greater protection of the lipids and proteins. Stress induced degradation of lipids and proteins decline the structural and functional integrity of cells and N supplementation assuaged the salinity mediated membrane degradation by preventing the generation of excess ROS. Earlier Ahanger and Agarwal [1, 21] have demonstrated reduced protease activity due to optimal supplementation of K, however reports discussing influence of N availability are not available. Stresses inflict plant metabolism by inducing protease [1, 21, 44] and lypoxygenase activity [45]. The oxidative signals are crucial for alleviating dormancy and quiescence, triggering cell cycle activation and the genetic as well as epigenetic control underpinning growth and differentiation responses under changing environmental conditions [51]. Redox signalling hub interacts synergistically with phytohormone network for growth regulation and modulations under stress [51, 52]. It was apparent that N availability imparted beneficial impact on the synthesis of secondary metabolites including phenols and flavonoids. Secondary metabolites including phenols and flavonoids are involved in regulation of auxin transport, photoprotection, mechanical support, seed dispersal and protection against insect herbivory [53]. Plants improve synthesis of flavonoids to protect the stress mediated redox unbalance-induced changes in metabolism, and in addition are involved in buffering the changes in ROS homeostasis for modulating the ROS-mediated signalling cascade [54]. Secondary metabolites including flavonoids prevent oxidative damage by inhibiting the formation of ROS and protect cellular functioning by scavenging radicals like superoxide [53]. Increased accumulation of phenolic compounds impart greater radical scavenging activity reflecting in apparent growth improvement under stressed conditions [1, 21]. Secondary metabolite compounds like chlorogenic acid, caffeic acid, quercitin and catechin accept electrons in apoplast thereby protecting the cell wall composition and also contribute enormously to the generation of ascorbate pool leading to improved functioning of AsA-GSH cycle [49]. PAL is key enzyme regulating the synthesis of secondary metabolites and in present study N mediated increase in its activity depicts the apparent influence of N on secondary metabolite accumulation. Non-enzymatic antioxidants neutralize ROS when enzymatic system becomes less efficient. It can be inferred from the present findings that increased N supplementation protected wheat seedlings against salinity mediated oxidative effects by up-regulating antioxidant and osmolyte metabolism, and secondary metabolite accumulation.

## Conclusion

Conclusively, increased N supplementation protects the growth and metabolism of wheat seedlings through up-regulation of the antioxidant system, osmolyte and secondary metabolite accumulation. N mediated maintenance of the redox homeostasis prevented ill effects of salinity on photosynthetic functioning. N at both levels proved beneficial in ameliorating the salinity triggered oxidative damage to significant extent. Antioxidant components, both enzymatic and non-enzymatic, increased due to N supplementation conferring its active involvement in their expression levels. Therefore making it evident that increasing N supplementation regulates salt tolerance in wheat through modulations in the metabolism of antioxidants, osmolytes and metabolites.

## Methods

### Experimental design, plant material and growth conditions

Wheat (*Triticum aestivum* L) seeds were procured from College of Agronomy Northwest A&F University Yangling Shaanxi, China. Seeds were sterilized using 0.01% HgCl_2_ followed by thorough washing with distilled water and were sown in bottom perforated pots filled with peat, compost and sand (3:1:1). Soil in pots was supplied with 0, 50 and 100 mg N kg^− 1^ soil in the form of urea. After 10 days of seedlings growth salinity stress was induced by applying 100 mM NaCl (100 mL per pot) on alternate days for 20 days. The native concentration of N, P and K in the soil was 65.98, 18.78 and 80.67 mg kg^− 1^ soil respectively with pH 7.57 and water field capacity 52.21%. Pots were arranged in complete randomized block design with five replicates for each treatment and were maintained under green house conditions at the College of Life Science, NorthWest A&F University Yaangling Shaanxi China. After fourty days of growth seedlings were analyzed for photosynthetic parameters, antioxidant and osmotic constituents, secondary metabolite accumulation and oxidative stress parameters.

### Estimation of pigments and photosynthetic parameters

Total chlorophyll and carotenoids were extracted by macerating fresh leaves in 80% acetone using pestle and mortar. Absorbance was recorded at 480, 645 and 663 nm against [55]. Photosynthetic efficiency, intercellular CO_2_ concentration and stomatal conductance were measured in fully expanded leaf using photosynthesis apparatus Li-6400 (LI-COR Inc., USA).

### Determination of leaf water content, soluble sugars, proline and glycine betaine content

Relative water content (RWC) of leaves was determined by following Smart and Bingham [56]. Content of free sugars [57, 58], free amino acids [59], free proline [60] and glycine betaine [61] were estimated in powdered dry samples in both treated and untreated samples.

### Estimation of hydrogen peroxide and superoxide

For estimation of H_2_O_2_, 100 mg fresh leaf tissue was homogenised in 5 mL of 0.1% trichloro acetic acid (TCA) and subjected to centrifugation at 10,000 g for 10 min. 500 μL supernatant was mixed with equal volume of potassium phosphate buffer (pH 7.0) followed by addition of 1 mL potassium iodide. After thorough mixing absorbance was recorded at 390 nm [62].For measurement of O_2_^−^ concentrations fresh tissue was homogenized in potassium phosphate buffer (65 mM, pH 7.8) and homogenate was centrifuged at 5000 g for 10 min. Supernatant was mixed with 10 mM hydroxylamine hydrochloride and left for 20 min followed by addition of sulfanilamide and naphthylamine. After 20 min of incubation at 25 °C absorbance was measured at 530 nm [63] and calculations were done using standard curve of NaNO_2_.

### Lipid peroxidation, lipoxygenase and protease activity

Lipid peroxidation was determined as content of malonaldehyde (MDA) formation. 100 mg fresh leaf tissue was macerated using 1% TCA followed by centrifugation at 10,000 g for 5 min. 1.0 mL supernatant was reacted with 0.5% thiobarbituric acid (4 mL) at 95 °C for 30 min and tubes were subsequently cooled on ice bath followed by centrifugation at 5000 g for 5 min. Absorbance of supernatant was measured at 532 and 600 nm [64]. Activity of LOX (EC 1.13.11.12) was estimated according to the method of Doderer et al. [65] and increase in absorbance was recorded at 234 nm using linoleic acid as substrate. An extinction coefficient of 25 mM^− 1^ cm^− 1^ was for calculation and expressed as units (1 μmol of substrate oxidized min^− 1^) mg^− 1^ protein. Protease (EC 3.4.21.40) activity was assayed by homogenizing fresh tissue in chilled 50 mM sodium potassium buffer (pH 7.4) containing 1% PVP and the homogenate centrifuged at 5000 g for 5 min at 4 °C. Tyrosine released was read at 660 nm after incubating 1 mL supernatant with casein at 40 °C and reacting the mixture with Folin Ciocalteu’s reagent in alkaline medium. Activity was expressed as μg tyrosine released mg^− 1^ protein [66].

### Determination of nitrate Reductase

For assaying nitrate reductase (NR) activity 300 mg fresh leaf tissue was incubated in 100 mM potassium phosphate buffer (pH 7.5) containing 200 mM KNO_3_ and 0.5% n-propanol (v/v) at 30 °C for 3 h in dark. Thereafter, aliquot (1 mL) was mixed with equal volume of 1% sulphanilamide and 0.2% 1-nephthylethylene diamine dihydrochloride, and mixture was allowed to stand for 20 min. Thereafter the absorbance was recorded at 540 nm [67].

### Assay of antioxidant enzymes

For extraction of antioxidant enzymes fresh 5.0 g leaf tissue was homogenised in pre-chilled pestle and mortar using phosphate buffer (100 mM, pH 7.8) containing 1% polyvinyl pyrolidine and 1 mM EDTA. The homogenate was centrifuged at 12,000 g for 15 min at 4 °C. For ascorbate peroxidase (APX) extraction buffer was supplemented with 2 mM ascorbate. Supernatant was used as enzyme source. Activity of superoxide dismutase (SOD, EC 1.15.1.1) was assayed by monitoring the ability of enzyme to inhibit the photochemical reduction of nitroblue tetrazolium chloride (NBT) at 560 nm [68] Assay mixture was incubated under florescent light for 15 min and the absorbance was recorded against the non-illuminated blank and activity expressed as EU mg^− 1^ protein. Catalase (EC 1.11.1.6) activity was assayed following Aebi [69] and change in absorbance was monitored at 240 nm for 2 min in an assay mixture containing 50 mM potassium phosphate buffer (pH 7.0), H_2_O_2_ and 100 μL enzyme extract. Extinction coefficient of 0.036 mM^− 1^ cm^− 1^ was used for calculation. Ascorbate peroxidase (APX, EC 1.11.1.11) was assayed by following method of Nakano and Asada [70] and disappearance of H_2_O_2_ was monitored as decrease in absorbance at 290 nm for 3 min. An extinction coefficient of 2.8 mM^− 1^ cm^− 1^ was used for calculation and activity was expressed as EU mg^− 1^ protein. For estimation of glutathione reductase (GR; EC 1.6.4.2) activity glutathione dependent oxidation of NADPH was monitored as change in absorbance at 340 nm for 2 min [71]. Activity was expressed as EU mg^− 1^ protein and extinction coefficient of 6.2 mM^− 1^ cm^− 1^ was used for calculation. Activity of MDHAR (EC: 1.6.5.4) was assayed in a reaction mixture containing Tris–HCl buffer (50 mM, pH 7.5), 200 μM NADPH, 250 μM AsA and enzyme. Absorbance was read at 340 nm for 2 min [72] and extinction coefficient of 6.2 mM^− 1^ cm^− 1^ was used for calculation. Activity of DHAR (EC: 1.8.5.1) was measured following Nakano and Asada [70] in assay mixture containing 50 mM potassium phosphate buffer (pH 7.0), GSH (2.5 mM), and 100 μM DHA. Change in optical density was read at 265 nm for 2 min and extinction coefficient of 14 mM^− 1^ cm^− 1^ was used for calculation.

### Estimation of ascorbate, reduced glutathione, and tocopherol

Ascorbate (AsA) content was determined by macerating fresh plant material in 6% TCA and supernatant was mixed with 2% dinitrophenylhydrazine and 10% thiourea. After incubating in water bath for 15 min samples were cooled and 5 mL of cooled 80% H_2_SO_4_ was added. Absorbance was taken at 530 nm [73]. Standard curve of AsA was used for calculation. Estimation of reduced glutathione (GSH) was carried by following method described by Ellman [74]. 100 mg fresh tissue was homogenised in phosphate buffer (pH 8.0) and 500 μL supernatant was mixed with 5, 5-dithiobis-2-nitrobenzoic acid. Optical density was read at 412 nm and concentration of GSH was determined from standard graph of GSH. Tocopherol was extracted in ethanol and petroleum ether (1.6:2). After centrifugation supernatant was incubated with 2% of 2, 2-dipyridyl in dark followed by addition o distilled water (4 mL). Absorbance was recorded at 520 nm [75] and standard curve was used for calculation.

### Estimation of total phenols, flavonoids and assay of phenylalanine ammonia lyase

Total phenols and flavonoids were estimated by following the method of Malick and Singh [76] and Zhishen et al. [77]. Phenylalanine ammonia lyase (PAL) was assayed following Zucker [78] and formation of trans-cinnamic acid was measured at 290 nm.

### Estimation of Na, K and N

Na and K were estimated flame photometrically described by Ahanger et al. [79]. Micro-Kzeldahl’s method suggested by Jackson [80] and modified by Iswaran and Marwaha [81] was employed for estimation of N content.

### Statistical analysis

Data is mean (±SE) of three replicates and for testing significance of data Duncan’s Multiple Range Test was performed using One Way ANOVA and least significant difference (LSD) was calculated at *p* < 0.05.

## Data Availability

All data generated or analysed during this study are included in this published article.
